# Supplementary material for: Stroke patient and stakeholder engagement (SPSE): concepts, definitions, models, implementation strategies, indicators, and frameworks—a systematic scoping review
Source: Syst Rev. 2024 Oct 31;13:271. doi: 10.1186/s13643-024-02686-y (PMC11526530; doi:10.1186/s13643-024-02686-y)
Supplement: Supplementary file 1 — Additional file 1. PubMed search strategy. [file 13643_2024_2686_MOESM1_ESM.docx]

Table 1: Search strategy of PubMed database

| database | Search strategy |
| --- | --- |
| PubMed | ("research"[MeSH Terms] AND ("Cerebrovascular Disorders"[MeSH Terms] OR "stroke"[MeSH Terms]) AND ("Patient Advocacy"[MeSH Terms] OR "Caregivers"[MeSH Terms] OR "Stakeholder Participation"[MeSH Terms])) AND (english[Filter]) |
